# *Portulaca oleracea* shows no ameliorative potential on ovariectomy-induced hormonal and estrous cycle dysregulation in normal cyclic rats: An experimental study

**DOI:** 10.18502/ijrm.v19i10.9822

**Published:** 2021-11-04

**Authors:** Izuchukwu Azuka Okafor, Uchenna Somtochukwu Okafor, Victor Ndukwe

**Affiliations:** ^1^Department of Anatomy, Faculty of Basic Medical Sciences, College of Health Sciences, Nnamdi Azikiwe University, Nnewi Campus, Nnewi, Nigeria.; ^2^Department of Obstetrics and Gynecology, Faculty of Clinical Sciences, College of Medicine, University of Ibadan, Nigeria.; ^3^Pan African University of Life and Earth Science Institute (Including Health and Agriculture) PAULESI, University of Ibadan, Ibadan, Nigeria.; ^4^Hematology Department, Babcock University Teaching Hospital, Ilisan Remo, Ogun State, Nigeria.

**Keywords:** Portulaca oleracea, Wistar rat, Ovariectomy, Estrous cycle, Hormones.

## Abstract

**Background:**

*Portulaca oleracea* (PO) is a widely known plant utilized for its medicinal attributes in the treatment of different illnesses.

**Objective:**

To investigate the effect of methanolic extract of PO (MEPO) on ovariectomy-induced reproductive toxicity in normal cyclic rats.

**Materials and Methods:**

Twenty 10-wk-old normal cyclic rats weighing 110-200 g were randomly divided into four groups (n = 5/group). Group A served as the control and received distilled water only. Group B was ovariectomized without treatment, while groups C and D were ovariectomized but treated with 400 and 800 mg/kg of MEPO, respectively, for 14 days. At the end of the experiment, body weight, serum hormonal levels, and estrous cycles were monitored across the groups.

**Results:**

Groups B, C, and D showed estrous cycle dysregulation and specific phase arrest when compared with the control. While a significant decrease in estradiol (p ≤ 0.001) and testosterone levels (p ≤ 0.001) were observed in groups B, C, and D, only groups C and D showed a significant increase in progesterone level when compared with the control (p ≤ 0.001, p = 0.01, respectively).

**Conclusion:**

The administration of 400 and 800 mg/kg MEPO is ineffective in ameliorating estrous cycle disruption and hormonal changes seen in ovariectomized normal cyclic adult Wistar rats.

## 1. Introduction

There is a growing tendency in the search for natural herbs with scientifically-proven therapeutic potentials especially in developing countries. Medicinal plants have continued to attract consideration in the global search for safe and effective herbal therapy for diseases affecting humans (1). *Portulaca oleracea* (PO) is a naturally occurring plant used in traditional and alternative medicine to treat different ailments. The World Health Organization has listed it as one of the most used medicinal plants, hence described with the term “Global Panacea” (2). The plant is used by herbalists in traditional medicine to treat snake and insect bites, as parts of this plant possess analgesic effects (3). Commonly called purslane, PO is considered the richest plant source of omega-3 fatty acids (2) and has been reported to also possess anti-aging (3), antioxidant, anti-inflammatory, anti-cancer, anti-microbial (4), immunological (5), and hepatoprotective properties (6). Several studies on reproduction have reported the effect of the administration of purslane including decrease in sperm motility and testosterone (TT) levels (7), sperm morphology distortions (8), and alteration of the estrous cycle (9).

Evaluation of the menstrual cycle (synonymous with the estrous cycle in rats) remains an indispensable measure of the normal reproductive state in humans. However, the short cycle length of rats makes it an ideal animal for studying changes during the reproductive cycle (10). Reproductive cycle disorders are associated with some infertility-related diseases in which ovariectomy (the surgical removal of the ovaries) is often carried out; for example, ovarian cyst, endometriosis, ectopic pregnancy, and pelvic inflammatory disease. It has been reported that about 10% of the world's female infertility cases require ovariectomy (11). The reproductive cycle is influenced by ovariectomy because the ovaries are chiefly responsible for the production of the female sex hormones - estrogen and progesterone (PG).

The normal estrous cycle is largely dependent on normal hormonal regulation. During the estrous cycle, prolactin, luteinizing hormone (LH), and follicle-stimulating hormone (FSH) remain low only to increase in the midday of the proestrus phase. The estradiol (E2) level increases at metestrous, reaching its peak during proestrus, and returning to baseline at estrous while PG secretion increases during proestrus and returns to baseline at estrous. PG secretion increases during metestrous and decreases during diestrus subsequently reaching its second peak near the end of proestrus while ovulation occurs from the commencement of proestrus to the end of estrous (12). The use of plant-based herbal products (including PO) to treat reproductive disorders even without knowing the physiological consequences of the treatment is a common practice, especially in poor-resource countries. This study utilized vaginal smear cytology to observe the estrous cycle in ovariectomized rats by the proportional measure of the three cell types seen in a vaginal smear: epithelial cell, cornified cell, and leukocyte, as described by Paccola and colleagues (10).

Our study aimed to unmask the potential role of the methanolic extract of PO (MEPO) in reducing the reproductive cycle abnormalities seen in ovariectomized adult Wistar rats.

## 2. Materials and Methods 

### Study setting

This experimental study was undertaken in the research laboratory of Anatomy Department of Nnamdi Azikiwe University between January-April, 2019.

### Plant collection, identification, and extraction

PO was harvested from marshy areas at Awka, Anambra State, Nigeria. PO was identified (ID/155A/P.oleracea) and authenticated in the botanical garden of the Department of Botany, Edo State University. A large amount of the collected plant was washed free of loam and aerial parts were separated from the roots and used for the phytochemical extraction. The aerial parts were air-dried for 2 wk and oven-dried at 40°C, to ensure complete dryness. The dried plant was ground, yielding about 500 g. The sample was marinated and extracted with 70% methanol (1:2 wt/vol B. Braun, Germany) for 72 hr at room temperature (26-28°C). The resulting solution was filtered and sieved; methanol was evaporated at a temperature of 40°C to give a yield of 10.2% of the starting material. The extraction was done as described by Okafor and co-workers (6).

### Animal source, care, and handling

Twenty normal cyclic female Wistar rats weighing 110-200 gr were selected for the experiment. The animals were confirmed to have a normal cycle through vaginal smear before being included for the study. Animals were obtained from the animal farm of the Faculty of Basic Medical Sciences, Nnamdi Azikiwe University, College of Health Science, Nnewi Campus, Nnewi, Nigeria. The Wistar rats were acclimatized for 2 wk, and were housed in standard cages: five rats per cage in a temperature-regulated room (25°C), with a 12-hr light/dark cycle, starting at 6:00 am. Standard laboratory rat chow and tap water were provided throughout the study.

### Vaginal smear cytology and estrous cycle evaluation

The estrous cycle of the animals was observed by vaginal smear cytology before sorting the animals into groups and consistently in the morning between 08:00 and 09:00 throughout the study. Vaginal secretion was collected by introducing the plastic pipette filled with 10 µL of normal saline (NaCl 0.9%, B. Braun, Germany) into the tip of the vagina for a suction-aspiration of the cells and mucus of the vagina. The collected sample was then placed on a slide and viewed under a light microscope (DM 750 Leica Microscope, Germany) with x10 objective lenses. The proportions of the three types of cells (round and nucleated: epithelial cells; irregular and anucleated: cornified cells; small and round: leukocytes) were used to determine the estrous cycle phase of the animals as documented in previous studies (10, 12). The vaginal smear was done by two experts independently and results were compared to avoid any subjective bias. Each animal was observed for two 4-day cycles to determine the regularity of the estrous cycle before being included in the study. Only 20 normal cyclic female Wistar rats were selected for this study. The vaginal smear was repeated after the administration of MEPO for 14 days to be able to compare with the initial smear cytology outcome for variations. Three animals per group were selected randomly for the vaginal smear. The photomicrography was done using the DM 750 digital Leica light microscope (Germany) computer software.

### Experimental procedure 

Twenty 10-wk-old adult normal cyclic female Wistar rats were divided into four groups (n = 5/each) based on their body weight. Group A served as the control group and received only distilled water. Rats in group B (OVX) were ovariectomized and given only distilled water. Rats in groups C (OVX 400) and D (OVX 800) were ovariectomized and received 400 and 800 mg/kg-1/day-1 MEPO, respectively, for 14 days. MEPO administration was done orally using oral gavage and based on the animal's body weight. The dosage and duration of administration were based on our previous study (6).

### Ovariectomy procedures

A bilateral ovariectomy procedure was carried out on all the animals in groups B, C, and D. The weights of the animals were taken with a digital weighing machine (Samurai Technoweigh, India) before the surgery, and the animals were anesthetized with 50 mg/ml of ketamine (Hameln Pharma Ltd., UK) administered intraperitoneally. The anesthetized animal was placed on its dorsal surface and the hair on the rat's abdomen was completely removed with a depilatory cream. The area to be operated was sanitized with ethanol, and a transverse peritoneal incision of 0.4-0.6 cm was made with a surgical scalpel (Natra, Germany) slightly toward the right on the mid-part of the abdomen (11).

To access the peritoneal cavity, adipose tissue was pulled away until the right uterine tube and ovary were identified. The left ovary was equally tracked through the same incision following the right uterine tube pathway. On identification of the ovary and uterine horn, a braided silk seam (Ethicon mersilk) was made around the area of the distal uterine horn, between the horn and the ovary. This junction was sectioned thereafter, removing the right and left ovaries. The uterine horn was returned to the peritoneal cavity after the removal of the ovaries. All of the ovariectomies carried out in this study were bilateral. The peritoneal incision was sutured in two strata (muscle and skin) using sterile sutures. The peritoneum and the muscle layers were sutured with an absorbable suture (Ethicon chromic sutures, US), while the skin was sutured with a non-absorbable suture (Ethicon mersilk sutures, US). Povidone-iodine was applied to the area to sanitize the skin after stitching. A high degree of hygienic procedures was sustained during the surgery. After the surgery, the rats were housed individually and provided with a clean and dry bedding made of 100% sterilized cotton fabric. This was done to provide extra comfort and to prevent hypothermia and/or possible contamination due to the constant, direct contact of the rats' ventral surface with the paddy husk bedding provided before the surgery (11). The animals had a 2-wk post-surgery recovery period.

### Animal sacrifice and blood sample collection

After the administration on the 14 ${}^{\mathrm{th}}$ day, all the animals from the groups were fasted overnight and euthanized the next day by cervical dislocation. Blood samples were collected by orbital puncture into appropriately labelled plain tubes. The blood was centrifuged and blood serum was extracted.

### Hormonal analysis

The collected blood was left to stand for 15 min in the sample container under room temperature before centrifugation at 1000 g for 5 min in a refrigerated centrifuge (Hunan Cenlee Scientific Instruments Co., Ltd. China) and the serum was extracted. Analysis for FSH, LH, PG, E2, and TT was carried out using AccuBind enzyme-linked immunosorbent assay (ELISA, Monobind Inc. Lake Forest, CA, USA) microwells for FSH, LH, PG, E2, and TT, respectively, with the product codes - 4925-300, 4825-300, 425-300, and 625-300) respectively.

### Ethical considerations 

The experimental procedures complied with Animal research: Reporting of in vivo experiments (ARRIVE) guidelines, National Institutes of Health guidelines, and the National Health Research Ethic Committee of Nigeria guidelines for the care and use of laboratory animals (13, 14). Animal health status was monitored throughout the experiment according to the Federation of European Laboratory Animal Science Associations guidelines (15). The ethical approval for this study was received from the Research Ethics Committee of the Anatomy Department, Faculty of Basic Medical Sciences, Nnamdi Azikiwe University, Nnewi Campus Nnewi, Nigeria (Code: AREC/ANA/2019/0015).

### Statistical analysis 

Data were analyzed using the IBM Statistical Package for the Social Sciences for Windows, version 23 (SPSS, IBM Corporation, Armonk, New York, USA). One-way analysis of variance (ANOVA), post-hoc LSD and student's *t* test were used to test for significance in changes seen in the variables across the groups. Tables were used for the representation of data, and values were considered significant at p < 0.05.

## 3. Results 

### Body weight measurement

There was an increase in body weight in groups B, C, and D. Significant weight gains were observed only in groups B (18.1%; p = 0.02) and D (44.5%; p = 0.03). The decrease in weight in the control group (-5.8%) was not significant (p = 0.05) (Table I).

### Estrous cycle examination

The estrous cycle across the study groups before and after the treatment with MEPO showed estrous cycle disruption and specific estrous phase arrest in both OVX- and MEPO-treated groups. The phase arrest observed in this study was at all four of the phases of the cycle (proestrus, estrous, metestrous, and diestrus) across the groups. The photomicrographs showing the estrous cycle phases for different animals in different groups were not shown due to a large number of slides; however, the estrous cycle was documented in Table II.

### Serum LH, FSH, E2, TT, and PG

There was no significant difference in the FSH and LH levels in the OVX, OVX400, or OVX800 groups when compared with the control group (p = 0.73 and p = 0.15, respectively). E2 and TT levels showed a significant decrease in the OVX, OVX400, and OVX800 groups when compared to the control group (p ≤ 0.001). Only OVX400 and OVX800 groups showed a significant increase in PG levels when compared with the control group (p ≤ 0.001 and 0.01, respectively) (Table III).

**Table 1:** The effect of MEPO on the body weight of ovariectomized adult female Wistar rats


**Groups **	**Pre-administration body weight (g)**	**Post-administration body weight (g)**	**% difference in body weight**
**A (control) **	170.00 ± 5.77	160.67 ± 3.76	-5.8
**B (OVX)**	155.00 ± 2.89	189.33 ± 7.06 ${}^{*}$	18.1
**C (OVX 400)**	136.33 ± 10.11	178.33 ± 4.41	23.6
**D (OVX 800)**	110.23 ± 9.92	198.67 ± 6.33 ${}^{*}$	44.5
Data presented as Mean ± SEM. Student dependent *t* test. Data were considered significant at p < 0.05. ${}^{*}$ P < 0.05, which indicates a significant difference between the pre- and post-administration body weights. The % difference in body weight change for each group was derived from the mean difference between pre- and post-administration body weights. OVX: Ovariectomized only, OVX400: Ovariectomized + 400 mg/kg MEPO, OVX 800: Ovariectomized + 800 mg/kg MEPO			

**Table 2:** The determination of the estrous cycle in ovariectomized and MEPO-treated ovariectomized adult Wistar rats


**Groups**		**R1**				**R2**				**R3**			
		**D1**	**D2**	**D3**	**D4**	**D1**	**D2**	**D3**	**D4**	**D1**	**D2**	**D3**	**D4**
**A (control)**													
	**Initial**	E	M	M	D	D	P	P	P	E	E	D	P
	**Final**	M	D	P	E	M	E	E	E	E	M	P	P
**B (OVX)**													
	**Initial**	M	D	D	P	E	E	E	E	M	E	P	M
	**Final**	E	P	E	E	E	M*	M*	M*	M	D*	D*	D*
**C (OVX400)**													
	**Initial**	D	D	P	E	E	M	M	D	D	D	E	M
	**Final**	M	P	E	P	D*	D*	M*	D*	P	P	D	D
**D (OVX800)**													
	**Initial**	P	E	E	M	D	D	D	P	P	P	M	D
	**Final**	P*	P*	P*	D	P*	P*	P*	P*	P	P	M	M
${}^{*}$ The cycle phases indicate several phases of estrous cycle arrest after 14-day MEPO administration in the ovariectomized groups. R1: First Wistar rat, R2: Second Wistar rat, R3: Third Wister rat, D1: First day, D2: Second day, D3: Third day, D4: Fourth day, Initial: Before MEPO administration, Final: After MEPO administration, P: Proestrus, E: Estrous, M: Metestrous, D: Diestrus, OVX: Ovariectomized only, OVX400: Ovariectomized + 400 mg/kg MEPO, OVX 800: Ovariectomized + 800 mg/kg MEPO													

**Table 3:** The effect of MEPO on serum LH, FSH, E2, TT, and PG


**Groups **	**LH (mIU/mL)**	**FSH (mIU/mL)**	**E2 (pg/ml)**	**TT (ng/ml)**	**PG (ng/ml)**
**A (control) **	2.1 ± 0.0	0.4 ± 0.0	152.0 ± 3.0	3.2 ± 0.0	2.3 ± 0.0
**B (OVX)**	1.8 ± 0.3	0.45 ± 0.1	96.5 ± 3.5 ** ${}^{a}$ **	0.2 ± 0.0 ** ${}^{a}$ **	2.7 ± 0.2
**C (OVX 400)**	2.0 ± 0.1	0.7 ± 0.1	96.0 ± 1.4 ** ${}^{a}$ **	0.2 ± 0.0 ** ${}^{a}$ **	4.3 ± 0.3 ** ${}^{a}$ **
**D (OVX 800)**	2.0 ± 0.2	0.8 ± 0.2	99.0 ± 4.0 ** ${}^{a}$ **	0.2 ± 0.0 ** ${}^{a}$ **	3.6 ± 0.1 ** ${}^{a}$ **
Data presented as Mean ± SD. One-way ANOVA, followed by multiple comparisons using LSD, ** ${}^{a}$ ** P < 0.05. LH: Luteinizing hormone, FSH: Follicle-stimulating hormone, E2: Estradiol, TT: Testosterone, PG: Progesterone, OVX: Ovariectomized only, OVX400: Ovariectomized + 400 mg/kg MEPO, OVX 800: Ovariectomized + 800 mg/kg MEPO					

## 4. Discussion 

This study aimed to evaluate PO's potential in ameliorating ovariectomy-induced hormonal and estrous cycle irregularity. The results showed a significant increase in body weight following the administration of MEPO in groups B and D when compared to the control. These findings are in line with a previous study, which reported a similar increase in body weight of animals following the administration of MEPO (6). It, however, does not corroborate the observations of another study, which reported a non-significant difference in body weight of animals treated with both aqueous and methanolic extract of PO (8). On the contrary, a study reported a significant decrease in body weight following the administration of PO (16). The opposing outcomes observed may be the result of varied study duration and dosage differences across the studies. There were no differences in the impact of ovariectomy and the administration of MEPO following ovariectomy on the animal body weight (Table I).

Several studies have investigated the effect of PO in different human tissues and systems, with some evidence portraying beneficial effects (4, 5, 7). These seemingly beneficial effects may have led to an increase in consumption and usage of this plant as medicine for different purposes. The implication of PO use in both male and female reproductive toxic effects has created the need for further evaluation of its effects and possible mechanisms (7, 9).

The regularity and formality of the estrous cycle are seen as a good measure of potential fertility. In this study, the observation of the estrous cycle across the study groups before and after treatment with MEPO showed estrous cycle disruption and specific-phase arrest in both the OVX- and MEPO-treated groups. This was different from the normal cycle observed in the control group (Table II). Out of the five animals used in each group for this study, three were randomly selected for the follow-up vaginal smear cytology and all of the selected animals showed either irregular cycle patterns or phase arrest (Table II). The phase arrest observed in this study was at all the cycle phases (proestrus, estrous, metestrous, and diestrus) across the groups (Table II). This is indicative of the reproductive toxicity associated with ovariectomy in the female reproductive system, as well as the ineffectiveness of MEPO in ameliorating such induced cycle disruption. This also suggests that MEPO may be implicated in the dysregulation of the reproductive cycle in animals as earlier published (9).

Of note, our study did not take the initial and final vaginal smear during a specific phase and period for each animal for more specificity but was only able to consider this at the group level. Hence, our study could not substantiate the specific impact of MEPO and/or ovariectomy on the different estrous phases. The current study also did not consider the impact of MEPO and/or ovariectomy on the duration of the estrous cycle as the animals for the study were selected based on the concurrent maintenance of a four-day normal cycle.

The hormonal analysis following ovariectomy as observed in this study showed a significant decrease in the TT and E2 levels when compared to the control (Table III). This finding was not any different after MEPO administration when compared to the control. This is in line with the findings of another study (7) which reported a significant decrease in TT levels following the administration of the extract. However, this finding does not align with another study (17) which demonstrated that hydroalcoholic (70%) extract (200 mg/kg) of PO had no significant effect on the E2 level in the serum of normal mice. Some human studies corroborate our findings: one of the studies (18) reported a decrease in TT and E2 after oophorectomy while the other (19) showed an association between oophorectomy and decreased TT levels. Low E2 serum level is indicative of menopause and/or ovarian failure, which thus supports the dysregulation seen in the estrous cycle. Interestingly, while the non-treated ovariectomized group showed no difference in its PG levels when compared to the other groups (Table III), both of the MEPO-treated ovariectomized groups showed a significant increase in the PG levels when compared with the control group (Table III).

The competitive affinity for the Phospholipase C - Protein Kinase C - signaling pathway (7) for E2 and PG could explain the increase in PG found in this study as E2 decreased. Our study also observed no significant difference in LH and FSH levels across the groups. FSH is responsible for menstrual cycle regulation and ovary stimulation for egg production through E2 stimulation. Abnormal changes in FSH could be indicative of ovarian toxicity such as ovarian cancer or polycystic ovarian syndrome. The observations of FSH and LH levels in this study are in accordance with another study that recorded similar outcomes after the administration of the ethanolic extract (200 mg/kg) of purslane (9). However, human findings showed an increase in FSH and LH levels after ovariectomy (18). Notwithstanding, our study established that ovariectomy or MEPO-treatment in ovariectomy had no significant effect on the FSH and LH levels.

## 5. Conclusion

This study has reestablished that ovariectomy causes a significant decrease in TT and E2 and also disrupts the estrous cycle in rats. More importantly, it has given clear evidence for the lack of potential of MEPO to repair ovariectomy-induced endocrine and estrous cycle damages. According to the findings, 400 and 800 mg/kg MEPO does not play any role in maintaining the normal estrous cycle and reproductive hormonal output in the absence of the ovaries.

##  Conflict of Interest

There is no conflict of interest to declare.
